# Alzheimer’s Disease and SARS-CoV-2: Pathophysiological Analysis and Social Context

**DOI:** 10.3390/brainsci12101405

**Published:** 2022-10-18

**Authors:** Genaro Gabriel Ortiz, Irma E. Velázquez-Brizuela, Genaro E. Ortiz-Velázquez, María J. Ocampo-Alfaro, Joel Salazar-Flores, Daniela L. C. Delgado-Lara, Erandis D. Torres-Sanchez

**Affiliations:** 1 Department of Philosophical and Methodological Disciplines and Molecular Biology in Medicine Service Hospital Civil, University Health Sciences Center, University of Guadalajara, Guadalajara 44340, Jalisco, Mexico; 2 Clinical Laboratory, UMF 61, Naucalpan 53000, State of Mexico, Mexico; 3 Geriatric Hospital-West General Hospital, SSA-Jalisco, Zapopan 45170, Jalisco, Mexico; 4 Department of Medical and Life Sciences, University Center of la Cienega, University of Guadalajara, Ocotlan 47820, Jalisco, Mexico; 5 Academic Department University Training Health Sciences, Autonomous University of Guadalajara, Zapopan 45129, Jalisco, Mexico

**Keywords:** Alzheimer’s disease, COVID-19, SARS-CoV-2, inflammation, neurodegeneration, social support, social behavior

## Abstract

The COVID-19 pandemic has proven to be a challenge for healthcare systems, especially in terms of the care of patients with Alzheimer’s disease (AD). Age is one of the major risk factors for severe forms of COVID-19, most probably due to the presence of comorbidities and inflammations. It is known that SARS-CoV-2 invades nerve endings and olfactory nerves through the binding of the spike protein to the angiotensin-converting enzyme 2 (ACE2) receptor. This interaction triggers an inflammatory cascade that results in cognitive impairment. In turn, the isoform of apolipoprotein-E4 (APOE-4ε) in AD is a risk factor for increased neuroinflammation through microglia activation, increased oxidative stress, and neurodegeneration. AD and SARS-CoV-2 are associated with increases in levels of inflammatory markers, as well as increases in levels of APOE-4ε, ACE2 and oxidative stress. Thus, there is a synergistic relationship between AD and SARS-CoV-2. In addition, the social isolation and other health measures resulting from the pandemic have led to a higher level of anxiety and depression among AD patients, a situation which may lead to a decline in cognitive function. Therefore, there is a need to develop strategies for keeping the patient calm but active.

## 1. Introduction

Alzheimer’s disease (AD) is a neurodegenerative condition that affects the central nervous system (CNS) with consequences on cognitive, behavioral, and functional attributes of the patient. Among the cognitive implications, memory loss, speech alteration, agnosia, apraxia, and changes in visuospatial functions stand out [1]. The key behavioral changes which may occur are depression, anxiety, anger, irritability, insomnia, and paranoia [2]. At the functional level, there are declines in the ability of the AD patient to independently perform daily activities such as eating, dressing, and cleaning [3]. Most cases of AD occur on a sporadic basis with a late onset after 65 years of age [4]. One risk factor for AD is increased expression of APOE 4ε [5]. On the other hand, early-onset AD associated with genetic mutations occurs less often [4]. The key mutations involve genes that encode the amyloid precursor protein (APP) and Presenilin 1 and 2. Hydrolysis of APP releases the neurotoxic peptide named amyloid beta (Aβ). In particular, the *hippocampus* is altered by deposits of Aβ, resulting in hyperphosphorylation of tau protein and association of aggregates known as neurofibrillary tangles. These neurofibrillary tangles affect neuronal signaling and lead to the death of the neurons [5,6]. It has been estimated that every year, 10 out of every 100,000 people develop dementia (mostly AD) worldwide. This figure represents 60 to 80% of cases of major cognitive disorder (MCD) in adults. Previous data indicate that from 1999 to 2019, there were increases in the incidence of AD-related death (16–30 deaths per 100,000 people), with a projected global increase in 175.6% by the year 2050 [7,8]. The pathogenesis of AD can be modulated by various modulators such as age, some comorbidities, environmental, biological, and genetic factors [9]. The biological factors have greater impact than the genetic factors due to the fact that they involve greater molecular deregulation. The environmental factor is related to the lifestyle of people. It has been reported that uncontrolled hypertension, obesity, hypercholesterolemia, and hyperglycemia may accentuate the progression of MCD [5,10]. Cardiovascular diseases and diabetes mellitus activate cellular inflammatory pathways which lead to increases in Aβ deposits and tau hyperphosphorylation [11]. In addition, alteration in insulin levels may affect the development of AD through its influence on synapse, inflammation, energy metabolism, and vascular function [12]. From another angle, increases in plasma and brain cholesterol by APOE 4ε affects the integrity of the Blood–Brain Barrier (BBB) by influencing its permeability, indicating a close relationship between AD and hypercholesterolemia [13]. Studies have shown that coronavirus disease 2019 (COVID-19) and AD share common risk such as age, sex, hypertension, diabetes, APOE 4ε, links with the ACE2 receptor, increased oxidative stress, and obesity [14,15,16,17].

The COVID-19 disease is caused by exposure to severe acute respiratory syndrome coronavirus 2 (SARS-CoV-2) which belongs to the *Coronaviridae* family. These viruses are characterized by the presence of a single-stranded RNA genome in the nucleus of each virion. Moreover, proteins known as spikes stand out on their nuclear envelopes. The viruses also contain membrane proteins, envelope proteins and accessory proteins. To initiate infection, the spike proteins bind to ACE2 receptors of the host cell through the S1 subunit. Then, the S2 subunit of the spike protein facilitates the fusion of the viral membrane with the host cell, subsequently leading to transcription and translation of the viral information in the cell [18,19]. The main symptoms of COVID-19 are fever, cough, shortness of breath, myalgia, fatigue, and in some cases, signs of pneumonia [20]. According to WHO data, as of September 2022, 6.5 million deaths from COVID-19 have been reported [21]. Data from a study in June 2022 show that, with more than 218 million infected people, Europe leads the other continents in the number of confirmed cases of COVID-19, followed by America with a figure of more than 160 million, and Asia with more than 135 million cases [22].

The major effects of COVID-19 infection are changes in respiratory function, which may be either moderate or severe. However, several studies have evaluated the effects of SARS-CoV-2 on the CNS, based on the presence of neurological symptoms such as loss of smell, taste, stroke, and severe neurological damage [15,23,24]. In one study, it was indicated that 36.4% of patients infected with severe COVID-19 presented with neurological manifestations such as headache, anosmia, and dysgeusia, which may be risk factors for cognitive impairment [15]. In support of this hypothesis, postmortem analysis has revealed the presence of SARS-CoV-2 viral RNA in the brains of infected patients [24,25].

In addition, age is a risk factor for the development of severe forms of COVID-19 disease. For example, in Italy, 9 out of 10 deaths were associated with individuals over the age of 70 [26]. It has been reported that more than 31.2% of the most severe cases of COVID-19 infection occurred in older adults with MCD and/or AD [27]. Also, it was found that seniors had a five-fold higher risk of morbidity and mortality than the general population [8]. Some possible reasons for the increase in vulnerability due to age factors include slow immune response, increase in basal level of inflammation, and the presence of diseases such as diabetes mellitus, cardiac, pulmonary, and neurodegenerative pathologies [28]. Apart from the vulnerability which increases due to age, patients with MCD and/or AD depend on caregivers for their survival and personalized clinical care. In the absence of these services, the risk of infection by SARS-CoV-2 increases, and the probability that the disease will advance to more severe stages is accentuated [26]. However, there are limited investigations aimed at elderly people with MCD and/or AD, with respect to the impact of these pathologies vis-a-vis SARS-CoV-2 infection, in addition to the impact of the vulnerable social context in which they find themselves. This analysis examines the resilience of the health system. Health is taken as an indicator of adaptability of the health sector in the face of the COVID-19 pandemic [29]. In the United Kingdom, during the evolution of the SARS-CoV-2 pandemic from 2020 to 2021, it was reported that elderly people and those with pre-existing medical conditions had higher risk of infection and mortality than the rest of the population, indicating a close relationship between social and biological phenomena. This report reflects an evidentiary gap regarding the COVID-19 pandemic which unfortunately is not exclusive to the United Kingdom [30]. Therefore, this study was aimed at analyzing the pathophysiology of the interaction between AD and SARS-CoV-2, as well as its social context.

We searched PubMed from March 1, 2020, to Octuber 1, 2022, and references from relevant articles available in English, using the search terms “SARS-CoV-2”, “Alzheimer Disease”, “brain”, “Major Cognitive Disease”,”COVID-19”, “vascular dementia”, “ACE2”, “APOE 4ε”, “neuroinflammation”, “oxidative stress”, “amyloid beta”, “tau”, “grey matter volume”, “lifestyle”, “exercise”, and “therapeutic”.

## 2. Effect of SARS-CoV-2 in AD Patients

There is a close relationship between potential pathogens and their repercussions on neurodegeneration and neuroinflammation, which may potentiate the development of AD. The SARS-CoV-2 infection leads to alteration in mental status, encephalopathy, MCD-like neurocognitive syndrome, psychosis, cerebrovascular events, development of neurodegenerative diseases, and worsening of clinical symptoms, especially in individuals with a previous diagnosis of AD [8,15]. Previous studies reported that cytomegalovirus and vesicular stomatitis viruses damage the neurons, thereby altering the release of neurotransmitters [16,31].

The SARS-CoV-2 has a high neuroinvasive potential. Thus, COVID-19-positive patients are likely to develop AD. Therefore, direct, and indirect mechanisms have been proposed with respect to how the SARS-CoV-2 may alter the CNS. Some of the proposed mechanisms involve thrombotic complications, increased inflammation, changes in blood pressure, and hypoxia [14,15]. It has been reported that patients with COVID-19 who developed severe stages of the disease presented with cognitive impairment and delirium, when compared to patients with less severe COVID-19 infection [32]. Evidence suggests that AD shares etiological factors with COVID-19 with respect to ACE2 receptors and increased pro-inflammatory markers [33]. It is important to note that SARS-CoV-2 invasion in neuronal tissues is regulated by the binding of the spike protein to ACE2 receptors from neurons, glial cells, and capillary endothelium [33].

SARS-CoV-2 stimulates reactive astrogliosis, microglial activation, and neuroinflammatory cascade; it thereby alters cortical and hippocampal function, resulting in neurological disorders and memory impairment [5,16,34]. Activation of microglia by SARS-CoV-2 infection triggers chronic inflammation and neurodegeneration. This damage may be exacerbated by increased expression of ACE2. Microglial activation and upregulation in the expression of ACE2 induce increased synthesis of nitric oxide (NO), which in large concentrations, is neurotoxic and contributes to the deterioration of AD [31]. *Postmortem* studies have shown that the expression of ACE2 in the brain of AD patients is increased, when compared to controls. In turn, the greater the expression of ACE2, the higher the levels of reactive oxygen species (ROS) and oxidative stress. This increase in ROS is exacerbated by SARS-CoV-2 infection which fuels the oxidative damage involved in the pathology of AD [14,33]. Moreover, evidence from animal models suggest that the *hippocampus* is highly susceptible to respiratory viruses. For example, in an animal model, it was demonstrated that the H5N1 virus has the potential to travel to the CNS of C57 black 6 Jax (C57BL/6J) mouse, resulting in increases in the release of pro-inflammatory cytokines, greater neuroinflammation, neuronal loss and protein aggregation [35,36].

From another perspective, it has been reported that viral infection influences loss of capacity to phagocytize Aβ peptide, which forms aggregates of plaques characteristic of AD pathology [16,37,38]. The cerebral hypoperfusion which occurs in some patients with SARS-CoV-2 also contributes to the accumulation of Aβ plaques [39]. Hypoperfusion is linked to hypoxia which contributes to neuroinflammation in AD patients. Moreover, cognitive dysfunction may result from ischemic damage and enhanced inflammatory response in the white matter of the limbic structure and *hippocampus*, key sites for the expression of enzymes involved in the inflammatory response, leading to deficits in neurocognitive processes [15,32].

Moreover, there is evidence of neuronal death after coronavirus infection through the olfactory bulb in animal models [16,40]. Recent human findings indicate that SARS-CoV-2 and the Middle East respiratory syndrome coronavirus (MERS-CoV) invade peripheral nerve terminals and access the CNS via synapses. A new study indicates that these viruses gain access to the brain through the olfactory nerves [8,31]. In neuroimaging studies of patients with COVID-19, it was observed that 34% of the participants presented with brain lesions attributable to SARS-CoV-2, with clear evidence of diffuse lesions of the subcortical and deep white matter, microhemorrhages, hemorrhages, and cerebral infarctions. However, there is need to corroborate these findings with other studies [15,16,39].

## 3. Effect of AD on the Severity of Patients with SARS-CoV-2 in Patients

**Figure 1:**
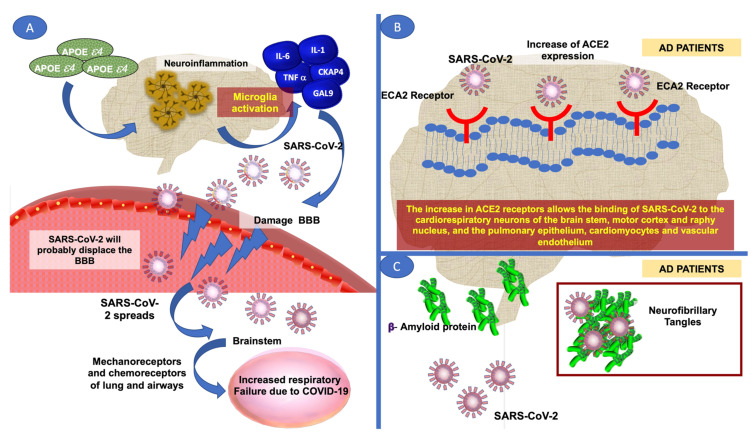
(**A**) APOE 4 is increased in patients with AD and with COVID-19. This increases neuroinflammation which damages the BBB, allowing the passage of viral particles such as SARS that can be distributed to mechanoreceptors and chemoreceptors in the lungs and airways. (**B**) AD patients have increased expression of ACE2, which increases the probability that SARS CoV-2 binds to its receptor in the respiratory epithelium, lung parenchyma, cardiomyocytes, vascular endothelium, cardiorespiratory neurons of the brainstem, motor cortex and raphe nucleus. (**C**) On the other hand, in patients with AD, the accumulation of amyloid beta plaques forms fibrils, allowing the deposition of viral particles, which increases the possibility of developing severe stages due to COVID 19 and higher mortality.

Recent data indicated that patients previously diagnosed with MCD and/or AD who later developed COVID-19 had higher morbidity and mortality due to SARS-CoV-2 infection, most likely due to increased levels of systemic inflammation. This exacerbated the damage caused by SARS-CoV-2, although other factors are being investigated, e.g., APOE 4ε [41,42,43]. The APOE 4ε is a key genetic risk factor, especially for AD [44]. It increases neuroinflammation and neurodegeneration through the activation of microglia, resulting in the release of Interleukin-6 (IL-6), Interleukin-1 (IL-1), cytoskeleton-associated protein 4 (CKAP4), Galectin-9 (GAL-9) and tumor necrosis factor-alpha (TNF-α) [5,8]. Data from previous investigations indicate an association between APOE 4ε and increased risk of severe COVID-19 infection [31,45]. Indeed, studies have shown that APOE 4ε triggers a storm of inflammatory cytokines which may contribute to a faster-debilitating and more severe SARS-CoV-2 disease [28,46]. Previous studies which analyzed AD patients who were positive for herpes simplex virus type I (HSV-I) found a higher frequency of APOE 4ε, with an odds ratio (OR) value of 16.8, indicating that APOE 4ε enhances susceptibility to viral infection [16,31,47]. Moreover, increased levels of APOE 4ε may accelerate damage to the BBB mainly to pericytes within blood vessels (Figure 1). It was shown that in patients with APOE 4ε, HSV-I was reactivated by events such as immunosuppression, peripheral infection, and inflammation, which are characteristic manifestations in patients with AD [31].

AD patients have a greater susceptibility to deteriorate to severe stages of the disease when infected with SARS-CoV-2, resulting in increased mortality; the extant literature highlights the analysis of IL-6, a common biomarker in AD and SARS-CoV-2 infection [5,32]. These studies showed that patients with severe stages of COVID-19 had elevated IL-6 levels, up to 2.9 times higher than the corresponding levels in those without complications. Similarly, AD is characterized by increased levels of IL-6 which translates into neuroinflammation and damage to mechanoreceptors and lung chemoreceptors [48]. In addition, AD patients have some risk genes such as oligoadenylate synthase 1 (OAS1), interferon alpha and beta receptor subunit 2 (IFNAR2) and coiled-coil alpha-helical rod protein 1 (CCHCR1). These genes are involved in interferon signaling, which contributes to raising the genetic risk associated with severe stages of AD due to increased inflammation in COVID-19 patients [33].

It has been proposed that AD patients have increased permeability of the BBB, thereby increasing the risk of the SARS-CoV-2 virus crossing and spreading from the olfactory nerve to the brainstem, and subsequently to mechanoreceptors and chemoreceptors of the lung and lower respiratory tract (Figure 1) [32,49]. This may account for the respiratory failure often seen in severe cases of COVID-19 [32,49]. AD patients have increased activity of ACE2, which has been identified as a risk factor for SARS-CoV-2 infection [31,33].

The SARS-CoV-2 virus is regulated by the binding of its glycoprotein to ACE2, and it is proposed that an increase in the expression of ACE2 in patients with AD favors the binding of SARS-CoV-2 to the respiratory epithelium, pulmonary parenchyma, cardiomyocytes, and vascular endothelium [31,50]. Moreover, ACE2 is distributed in central areas of the brain, e.g., cardiovascular neurons, cardiorespiratory neurons of the brainstem, motor cortex, and raphe nucleus. In other neuronal cells, ACE2 is expressed when the microglia undergo activation [31]. In AD patients, a positive correlation has been reported between increased ACE2 and oxidative stress, especially increased carbonylated proteins and oxidation of peroxidexin 6, the enzyme responsible for the antioxidant activity of peroxidase [51].

Another mechanism that explains how AD contributes to the severity of COVID-19 involves the accumulation of Aβ plaques. These plaques form a network of fibrils that allow the depositing of viral particles, thereby increasing the patient’s immune response to SARS-CoV-2 (Figure 1) [8]. Table 1 shows a total of 21 published studies on the impact of SARS-CoV2 on patients with AD and *vice versa*, demonstrating a vicious circle. In this Table, COVID patients and AD patients share a high inflammatory response, high expressions of APOE4ε and ACE2, and high levels of oxidative stress.

## 4. Patients with AD in the COVID-19 Pandemic

The COVID-19 pandemic has significantly complicated the search and follow-up of patients affected by AD. The impossibility of continuing to control the different existing pathologies and isolation, especially among the elderly, has increased the risk of the appearance of AD and aggravated the symptoms of loss of orientation. Social factors such as isolation, poor support networks, and limited access to physical or mental health services, lead to patients’ stress, hyperactivation of the pituitary–hypothalamic–adrenal axis, chronic inflammation, cytokine storm, and oxidative stress, all of which may cause alterations in mental health [16,59].

The principles of prevention and protection are fundamental for older people with MCD or AD. Increased morbidity and mortality are expected in this group of patients, taking into account that the risk of contagion by COVID-19 is increased by failure to follow recommendations of public health authorities with respect to hand hygiene, covering the mouth and nose when coughing, monitoring and reporting symptoms of COVID-19, and maintaining physical distance [8,26,34,60]. Since 2019, MCD patients and their loved ones have been under great distress, especially from the COVID-19 pandemic. In order to live at home as independently as possible, many patients rely on the daily help of their loved ones for their physical and mental well-being, and for provision of the best possible quality of life: they need daily routines, regular contact, and physical proximity to those close to them, whether they live at home or in a long-term care facility [8,32].

However, during the pandemic, contact opportunities were limited or even impossible due to temporary restrictions on visits and quarantines [59]. To ensure the best possible protection against the risk of infection, the needs of people with MCD have often been pushed into the background or unrecognized [8]. Concern for loved ones, isolation, and loneliness have been constant worries and psychological suffering for patients and their families. In many cases, psychiatric disorders secondary to MCD are increased, with consequent deterioration in health [61].

In a study conducted on 40 patients diagnosed with AD and mild amnesic cognitive impairment during five weeks of COVID-19 confinement, significant increases in levels of agitation, apathy, and impaired motor activity were reported [62]. These results were associated with the interruption of the care programs in which the patients participated, as well as changes in routine in their daily activities [62]. Another study conducted on 38 AD participants during the confinement revealed that 26.32% of participants manifested neuropsychiatric changes [59]. In addition, the duration of confinement was positively correlated with the severity of neuropsychiatric symptoms in the patients and distress experienced by their caregivers, possibly due to the reduction in social contact and the lack of stimulating activities. The longer the confinement, the more severe the neuropsychiatric symptoms in the AD patients [59].

It should be noted that due to the restrictions imposed by COVID-19, there was indefinite cancellation of elderly care programs during the day or nursery, as well as food programs; home care services by specialists, among other supports and health services, were canceled indefinitely. Thus, family members at home ended up being solely responsible for the care of these patients, a situation which increased the burden of complex medical tasks such as tube feeding, colostomy care, catheter care, and injections, among others, leading to frustration, stress, depression, and burnout in caregivers [63].

Studies indicate that 50% of older adults were at home with family care during the pandemic, while the rest were in nursing homes or help houses. The main reasons why older adults were referred to help homes were: (a) the need for specialized care (65%), (b) health of the caregiver (49%), and (c) increase in cognitive impairment (46%). In the United States of America, the government requires caregivers to take a 75-hour course prior to obtaining their accreditation. In Germany, at least 50% of the staff are required to have three-year experience in the field. In contrast, in other countries, the requirements are minimal, and usually caregivers are people without technical skills. Thus, with the passage of time, these untrained caregivers easily experience burnout. This also means that the patient who is at home or in an institution may have feelings of loneliness, helplessness, and boredom during the confinement due to lack of stimulatory activities, limited social interaction, changes in psychological accompaniment or even the lack of medical attention itself. Therefore, it is essential to develop protective strategies for patients with AD who are at home with family members, and for those who are housed in help homes [64,65,66]. Some strategies proposed for improved places of residence for older adults are described in Table 2.

The content of this chart is based on the article by Olson & Albensi [66].

**Figure 2:**
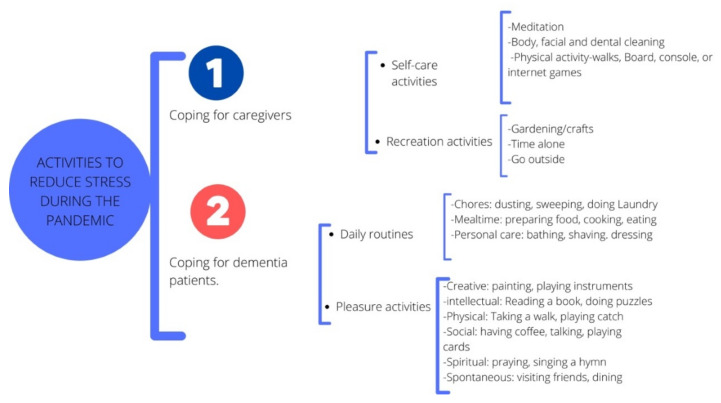
Activities to reduce stress during the pandemic. The design of the image is own creation, but the information of the image was based on recommendations to develop activities in the house of the Alzheimer’s Association and in the article of Bacsu [63,67].

It is important to limit the risks of contagion by SARS-CoV-2 through the strict application of barrier measures and the empowering of families and the public to protect vulnerable people [59,60]. However, it is also important to encourage people living with AD to remain active and continue as much as possible with routines that favor their quality of life. It has been suggested that patients with AD should engage in activities that provide them a sense of well-being to help reduce the level of anxiety or restlessness which may be experienced while being inactive [8]. Various activities can be carried out with a person living with AD, but in a situation of confinement, caregivers should opt for activities done at home, especially those that integrate creativity [60]. To avoid situations of frustration or rejection, it is important to adjust the activities to the real capabilities and the degree of satisfaction that the patient with AD may feel. In addition, the activities should be clearly explained to the patient in order to encourage them and to combat apathy. Creating a routine for patients with MCD is a fundamental factor in providing them with a safe and stable environment during periods of confinement [62]. It is also essential to give the AD patient the opportunity to express their concerns and to help them understand situations arising from confinement. In addition, the need to set up a daily routine that allows people with AD a serene, calm, and organized environment at home, has been recommended [67]. Figure 2 shows activities proposed for reduction in stress for caregivers and patients with MCD.

As shown in Figure 2, physical activity is fundamental for a healthy lifestyle, and several meta-analyses have demonstrated the benefits of physical exercise on physical function, as well as benefits for behavioral and neuropsychological symptoms in patients with MCD [68]. However, some barriers such as a lack of access to activities that encourage physical exercise, difficulty in moving to community sites where exercise classes are offered, and limited caregiver support, may arise in this population. The pandemic added even more limitations and challenges. Therefore, a search for alternatives for maintenance of minimum physical activity is proposed. If the AD patient continuously exercises outdoors, staying home may backfire. On the contrary, if the patient is not used to indulging in physical activity, it is recommended that they start with a light exercise adapted in intensity and duration, taking into account their physical and general conditions. It is important to avoid exercise that may cause discomfort or pain, and to stop the session if the patient is uncomfortable [69].

It should be noted that technology and social networks are necessary tools for improving the quality of life during confinement, since they are useful in maintaining contact with loved ones and facilitating remote interaction through video conferencing or phone calls. In addition, online exercise programs (exergames) and virtual reality games have been developed, wherein the patient can synchronously and asynchronously follow a series of exercises through the computer, tablet, phone, or console, thereby improving their physical and emotional well-being [70]. Finally, telemedicine rebounded as a new tool due to the COVID-19 pandemic, and technology was used in some communities to remotely follow patients with MCD or AD. Indeed, it has been reported that older adults with AD accepted the use of video calls with high degrees of satisfaction [71,72,73].

## 5. Conclusions

This study reveals that there is a close relationship in the pathophysiology between SARS-CoV-2 and AD. Data show that severe stages of COVID-19 may contribute to the development of neurodegenerative diseases, to some extent, and through certain mechanisms. The SARS-CoV-2, a virus with high neuro-invasive potential, penetrates nerve endings and olfactory nerves via the binding of its spike protein to the ACE2 receptor, thereby triggering the inflammatory cascade associated with cognitive impairment, neuroinflammation and neurodegeneration. Patients diagnosed with AD may progress to more severe stages due to SARS-CoV-2 infection, as a result of increase in APOE 4ε expression and increased activity of ACE2 in these patients. These factors increase the susceptibility to viral infection, enhance the permeability of BBB, increase inflammation and immunosuppression, thereby generating a suitable environment for viral proliferation. The social context is indispensable for reduction in the risk of SARS-CoV-2 infection. Therefore, it is important to improve the places of residence of AD patients, and to enhance activities aimed at reducing stress and increasing their quality of life.

## Figures and Tables

**Table 1:** Studies assessing the effect of SARS-CoV-2 in AD patients and *vice versa*.

Type of Study	n	Principal Findings and Characteristics of the Study	Reference
Review	NAP	**Pathophysiological response** Inflammation: increased rates of IL-6, IL-1 and GAL-9 and 3 in patients with COVID-19 and AD. In contrast, CKAP4 was identified as elevated only in patients with COVID-19. The increase in inflammation was probably related to the increase in mortality due to COVID-19 and the progression of AD by increasing the production of Aβ plates. An increase in GAL-9 and 3 can make it easier for SARS-CoV-2 to enter the lungs. APOE 4 ε: increased expression is reported in patients with COVID-19 and AD, and this may be associated with increased risk of severe stages of COVID-19; allele 4ε increases CNS fibrinogenesis in AD. ACE2: high levels are reported in patients with COVID-19 and AD, which may indicate an increased risk of developing these co-morbidities. Oxidative stress: increased NO production in patients with both co-morbidities.	[5]
		**Study specifications** Statistical data: NAP; Progression stage of patients with AD: NAP; Age range of participants: NAP; Long-term effects: NA.	
Review	NAP	**Pathophysiological response** Inflammation: increased rates of IL-6 in patients with COVID-19 and AD. IL-6: positively correlates with the severity of COVID-19, which can have an impact on neurodegeneration. APOE 4 ε: increased expression is reported in patients with COVID-19 and AD. Increased expression of this allele is associated with immunosuppression in patients with SARS-CoV-2 and increased risk of AD. ACE2: high levels are reported in patients with COVID-19 and AD. Highly expressed in patients with AD results in a higher rate of invasion and distribution of SARS-CoV-2. Oxidative stress: increased NO production in patients with both co-morbidities. Elevated concentrations of NO can affect neurotoxicity.	[31]
		**Study specifications** Statistical data: NAP; Progression stage of patients with AD: NAP; Age range of participants: NAP; Long-term effects: NA.	
Review	NAP	**Pathophysiological response** Inflammation: the increased levels of IL-6 and TNFα in patients with COVID-19, increased the hyperphosphorylation of the Tau protein and caused the accumulation of plaque Aβ and impaired memory. APOE 4 ε: increased expression is reported in patients with COVID-19 and AD. APOE 4 ε increased risk of infection and mortality by COVID-19 and genetic risk factor of AD. ACE2: high levels are reported only in patients with COVID-19. High expression in neurons and glial cells, possibly associated with neuronal death from SARS-CoV-2 infection.	[37]
		**Study specifications** Statistical data: NAP; Progression stage of patients with AD: NAP; Age range of participants: NAP; Long-term effects: NA.	
Review	NAP	**Pathophysiological response** Inflammation: IL-6 levels have increased in patients with COVID-19, which could exacerbate the damage to BBB and neural and glial cells with long-term sequelae. Microglia loses its ability to phagocyte plaque Aβ. APOE 4 ε: increased expression is reported in patients with COVID-19 and AD, its net increase can promote vulnerability to viral infection. ACE2: high levels are reported only in patients with COVID-19, may be associated with damage to neurons.	[16]
		**Study specifications** Statistical data: NAP; Progression stage of patients with AD: NAP; Age range of participants: NAP; Long-term effects: NA.	
Letter to editor	NAP	**Pathophysiological response** Inflammation: In general, the authors only mention an increase in inflammatory response in patients with COVID-19 and AD. APOE 4 ε: increased expression is reported in patients with COVID-19 and AD. ACE2: high levels are reported in patients with COVID-19 and AD. No additional information is provided for inflammation, APOE 4 ε or ACE2.	[15]
		**Study specifications** Statistical data: NAP; Progression stage of patients with AD: NAP; Age range of participants: NAP; Long-term effects: the author suggests that it is important to evaluate patients who had long-term COVID-19 infection to know the neurological repercussions.	
Review	NAP	**Pathophysiological response** Inflammation: increased rates of IL-6, IL-1β, IL-12, GAL-3 and TNFα in patients with COVID-19 and AD. Systemic inflammation induces activation of microglia and astrocytes, releasing pro-inflammatory cytokines associated with AD and COVID-19 patients. For GAL-3 elevated levels occur in severe COVID-19 and AD patients, promoting Aβ aggregation. APOE 4 ε: increased expression is reported in patients with COVID-19 and AD, and this is a risk factor for AD and SARS-CoV-2, particularly for developing severe stages. ACE2: high levels are reported in patients with COVID-19 and AD; the increased expression may be a risk factor for SARS-CoV-2 infection mediated by increased oxidative stress. Oxidative stress: increased ROS production in patients with both co-morbidities.	[14]
		**Study specifications** Statistical data: NAP; Progression stage of patients with AD: NAP; Age range of participants: NAP; Long-term effects: NA.	
Systematic review and meta-analysis	NAP	**Pathophysiological response** Inflammation: increased rates of IL-6 in patients with COVID-19. In all studies analyzed, elevated levels were found, particularly in patients with severe evolution due to SARS-CoV-2, as the level of IL-6 was positively correlated with bilateral lung damage (r = 0.45, P = 0.002).	[48]
		**Study specifications** Statistical data: NAP; Progression stage of patients with AD: NAP; Age range of participants: NAP; Long-term effects: NA.	
Commentary	NAP	**Pathophysiological response** Inflammation: the authors generally report that a storm of inflammatory cytokines is released in patients with COVID-19. APOE 4 ε: this study shows increased expression for patients with COVID-19. It is reported at a high number of copies predisposing to the development of a more severe stage by SARS-CoV-2. No other information is provided for ACE2 or oxidative stress.	[46]
		**Study specifications** Statistical data: NAP; Progression stage of patients with AD: NAP; Age range of participants: NAP; Long-term effects: NA.	
Review	NAP	**Pathophysiological response** Inflammation: increased rates of IL-6, IL-1β, IL-17 and TNFα in patients with COVID-19 and AD. The increased cytokines damage different regions of the brain. APOE 4 ε: increased expression is reported in patients with COVID-19 and AD, it is an allele that increases the risk of developing AD. ACE2: high levels are reported in patients with COVID-19 and AD; greater expression increases the probability of binding with SARS-CoV-2 and its permeability through the BBB. Oxidative stress: increased ROS production in patients with both co-morbidities.	[8]
		**Study specifications** Statistical data: NAP; Progression stage of patients with AD: NAP; Age range of participants: NAP; Long-term effects: NA.	
Letter to editor	451.367	**Pathophysiological response** APOE 4 ε: increased expression is reported in patients with COVID-19 and AD; there is an increase of 14 times more risk of developing AD in European population. The APOE4ε allele increases the risk of serious COVID-19 infection, independent of pre-existing dementia. No other information is provided for inflammation, ACE2 or oxidative stress.	[45]
		**Study specifications** Data from the participants and the study: age range between 48 and 86 years; of genetically European descent, the sample size of 451,367 corresponds to 90% of the sample of reference centers in England. A logistic regression model was used.	
Retrospective cohort study	G1: 19G2: 23	**Pathophysiological response** ACE2: high levels are reported in patients with COVID-19; the authors report a reduction in the ACE2 receptor in patients with AD and propose a decrease in hospitalization time for patients with COVID-19 and AD. No other information is provided for inflammation, APOE 4 ε or oxidative stress.	[34]
		**Study specifications** Data from the participants and the study: This study included 19 patients with AD (G1, Group 1) and 23 patients without AD (G2, Group 2), both from groups with a diagnosis of COVID-19. Participants have an age range of 65 to 100 years, with a value of Minimental >19 points, and patients with other neurological diseases and psychiatric diseases were excluded.	
Original	2547	**Pathophysiological response** Inflammation: increased rates of TNFα and IFNγ in patients with AD. Systemic inflammation leads to increased proinflammatory cytokines associated with more serious phases of COVID-19 and AD. No other information is provided for APOE 4 ε, ACE2 or oxidative stress.	[33]
		**Study specifications** Data from the participants and the study: 2547 DNA samples were collected from different UK research institutes of the UK Alzheimer Research Network, which were divided into 1313 AD cases and 1234 controls.	
Postmortem case series	43	**Pathophysiological response** Inflammation: SARS-CoV-2 is linked to an increase in cytokine storm, resulting in neuroimmune stimulation and systemic damage. No other information is provided for inflammation. ACE2: high levels are reported in patients with COVID-19, and it had a higher expression in oligodendrocytes. No additional information is provided for APOE 4 ε, or oxidative stress.	[24]
		**Study specifications** Data from the participants and the study: Participants were 51 to 94 years old; inclusion criteria included a positive diagnosis of COVID-19; 16 were female and 27 were male. 13 participants had pre-existing neurologic conditions, with viral pneumonia as the cause of death.	
Commentary	NAP	**Pathophysiological response** Inflammation: increased levels of IL-1 and IL-6 in patients with COVID-19 and AD. Both patologies activate the microglia and stimulate an increase in the cytokine storm. No other information is provided for APOE 4 ε, ACE2 or oxidative stress.	[28]
		**Study specifications** Statistical data: NAP; Progression stage of patients with AD: NAP; Age range of participants: NAP; Long-term effects: NA.	
Review	NAP	**Pathophysiological response** Inflammation: increased rates of NLRP3, IL-1β, IL-6, IL-17 and TNFα in patients with COVID-19 and AD. NLRP3 stimulates the ORF3 protein of the SARS-CoV-2 virus, which induces inflammatory activation leading to unregulated hyperinflammation, at the same time increasing the production of IL-1β induces neuroinflammation, neural death and cognitive deficiency involved in the pathogenesis of AD. ACE2: high levels are reported in patients with COVID-19 and AD; this study agrees with other authors that ACE2 receptor increases and oxidative stress increases. Oxidative stress: increased ROS production in patients with both co-morbidities. No other information is provided for APOE 4 ε.	[52]
		**Study specifications** Statistical data: NAP; Progression stage of patients with AD: NAP; Age range of participants: NAP; Long-term effects: NA.	
Perspective article	NAP	**Pathophysiological response** Inflammation: increased rates of TNFα, IL-6, IL-8, IL-10 and NLRP3 in patients with COVID-19 and AD. Increased levels of inflammation are associated with mortality and severe progression of SARS-CoV-2 and its responsiveness to infection control. Additionally, chronic inflammation is linked to the onset of AD. No additional information is provided for inflammation. No other information is provided for APOE 4 ε, ACE2 and oxidative stress.	[53]
		**Study specifications** Statistical data: NAP; Progression stage of patients with AD: NAP; Age range of participants: NAP; Long-term effects: NA.	
Review	NAP	**Pathophysiological response** Inflammation: increased rates of IL-1 IL-6, TNFα and IFNγ in patients with COVID-19 and AD. SARS-CoV-2 activates the procapoptotic pathway through hyperinflammation and a cytokines storm that can trigger a systemic inflammatory response. Regarding Alzheimer’s, it is unclear if neuroinflammation is an underlying cause or if neuroinflammation contributes to the development of Alzheimer’s. APOE 4 ε: increased expression is reported in patients with COVID-19 and AD, and APOE 4 ε is a higher-risk gene of AD that may be implicated in severe SARS-CoV-2 infection. ACE2: high levels are reported in patients with COVID-19 and AD. High ACE2 synthesis in patients with AD may facilitate the invasion of SARS-CoV-2 into the CNS and speed up viral transmission. Oxidative stress: increased ROS production in patients with both co-morbidities.	[54]
		**Study specifications** Statistical data: NAP; Progression stage of patients with AD: NAP; Age range of participants: NAP; Long-term effects: NA.	
Review	NAP	**Pathophysiological response** Inflammation: increased rates of IL-6, IL-10, and TNFα in patients with COVID-19 and AD. A heightened inflammatory response affects neural damage and induces neurological changes. APOE 4 ε: increased expression is reported in patients with COVID-19 and AD. APOE 4 ε increases the risk of COVID-19 infection. ACE2: high levels are reported in patients with COVID-19 and AD. This increase has an impact on vulnerability to infiltration and SARS-CoV-2 infection. Oxidative stress: increased ROS production in patients with both co-morbidities.	[55]
		**Study specifications** Statistical data: NAP; Progression stage of patients with AD: NAP; Age range of participants: NAP; Long-term effects: NA.	
Commentary	NAP	**Pathophysiological response** APOE 4 ε: increased expression is reported in patients with COVID-19 and AD. This is a variant that increases the risk not only of developing AD, but also of being susceptible to SARS-CoV-2 infection. ACE2: have been missed in this study. No other information is provided for inflammation and oxidative stress.	[56]
		**Study specifications** Statistical data: NAP; Progression stage of patients with AD: NAP; Age range of participants: NAP; Long-term effects: NA. The authors worked with a MetaCore analysis, with a sample size of ten patients with AD, in a cohort study.	
Review	NAP	**Pathophysiological response** Inflammation: increased rates of IL-6, TNFα, IL-1β, IL-8 and NLRP3 in patients with COVID-19 and AD. The authors suggest SARS-CoV-2-induced hyperinflammatory status. ACE2: high levels are reported in patients with COVID-19 and lower rates in patients with AD. For this study, the authors suggest lower levels of ACE2 in CNS tissues, such that it is assumed that SARS-CoV-2 generates little CNS inflammation. Oxidative stress: increased ROS production in patients with both co-morbidities. The authors support other studies described here about oxidative levels. No other information is provided for APOE 4 ε.	[57]
		**Study specifications** Statistical data: NAP; Progression stage of patients with AD: NAP; Age range of participants: NAP; Long-term effects: NA.	
Review	NAP	**Pathophysiological response** Inflammation: increased rates of IL-2, IL-6, IL-15, and TNFα in patients with COVID-19 and SNC. In this study, SARS-CoV-2 is reported to increase pro-inflammatory status within the CNS. ACE2: high levels are reported in patients with COVID-19 and lower rates in SNC. In this study, the high number of ACE2 receptors facilitates entry to SARS-CoV-2, and the authors suggest that the distribution of receptors in the CNS is not enough to describe viral neurotropism. Oxidative stress: increased ROS production in patients with COVID-19. The authors support the oxidative response described in other studies included in this table. No other information is provided for APOE 4 ε.	[58]
		**Study specifications** Statistical data: NAP; Progression stage of patients with AD: NAP; Age range of participants: NAP; Long-term effects: NA.	

IL, interleukin; CKAP4, cytoskeleton-associated protein 4; GAL-9, galectin 9; TNF, tumor necrosis factor; APOE, apolipoprotein E; ACE2, angiotensin-converting enzyme 2; NO: nitric oxide; ROS, reactive oxygen species; IFN, interferon; NLRP, nucleotide-binding domain and leucine-rich repeat pyrin domain containing 3 inflammasome. NAP: Not applicable; NA: Not available. The bold highlights the Pathophysiological Response and the conditions under which the study was conducted. Additionally, Pathophysiological Response is subdivided into the categories that are underlined.

**Table 2:** Characteristics to improve in nursing homes.

Area	Improvement
Lighting	Expose the older adult at least 2–3 h at 1000 lux to reduce agitation and improve sleep. Use designs that reduce confusing shadows and glare: install anti-reflective glass in bedrooms, bathrooms, and kitchens. Use brighter ambient light in the blue spectrum in common areas and entrances to outdoor spaces; use amber light at night to aid in care. Remove bright lighting above the bed, use strip lighting around doors and floors leading to the bathroom, and use window coverings to reduce glare from bright moonlight at night.
Colors	The use of contrasting colors and tones can help differentiate objects, define edges, help patients find their way, prevent trips and falls, reduce anxiety, and promote independence: Blue spectrum colors: help create a sense of calm and make the room size appear larger and fresher Green: is associated with nature, can reduce central nervous activity, calm and relax, can also make the room seem bigger and fresher. Red and fall colors: can stimulate the production of adrenaline and make the room feel smaller and warmer. Avoid very dark or pastel colors; use matte finishes, smooth and without black lines or circles.
Signage	The use of patient maps to help guide the patient is paramount; it must be cognitively easy to handle, with a precise sequence, and with an appropiate level of stimulation. Guidance involves building design, signs, labels, pictures, and personal items on doors. The use of a small-scale group design (10–12 residents per household) is recommended. Spatial design that provides a continuous route (circular or L-shape routes) and visual cues at each change in direction, eliminates hallways where possible, and centralizes kitchen/dining/living area; use of murals to conceal exit doors from the interior; access to bathrooms from rooms, access to outdoor spaces.
Outdoor spaces	Access to gardens and outdoor spaces will improve socialization, mental health, efficiency, and sleep duration. The results may be due to reminiscence and sensory stimulation. Spaces should be safe, unobstructed, and unlocked from inside; non-slip, non-glossy driveway, gentle slopes, raised fences, hidden outside doors; textures and shapes of materials that provide an experience of touch and surface continuity. Use of aromatic, non-toxic and, low plants near windows that do not hinder the interior view of the space; potted plants placed near seating areas; drinking water features to encourage calm, handrails, shade trees to sit on, furniture to sit on, adequate lighting for night visits, and open spaces for picnics and other activities.
Others	Reduction in loud noise (alarms and advertisements) to avoid overstimulation. Background music to reduce boredom. Control room temperature and have a ventilation system that reduce air currents, cold spots, and spreading respiratory diseases. Home decor, including art. Furniture and personal items that provide family comfort. Private bedroom focused on the preferences of the patient. Spaces for recreational activities: music therapy, arts, crafts, games, reading, exercise. Use of SMART technologies.

## Data Availability

Data are available on request from the authors.
